# What are patients’ expectations about the organization of their primary care physicians’ practices? 

**DOI:** 10.1186/s12913-015-0985-y

**Published:** 2015-08-14

**Authors:** Paul Sebo, François R. Herrmann, Patrick Bovier, Dagmar M. Haller

**Affiliations:** Primary Care Unit, Faculty of medicine, University of Geneva, Geneva, Switzerland; Geriatrics Division, Department of Internal Medicine, Rehabilitation and Geriatrics, Geneva University Hospitals, Geneva, Switzerland; Primary care practice, Lausanne, Switzerland; Primary Care Unit, Faculty of medicine, University of Geneva, Geneva, Switzerland; Department of Community, Primary Care and Emergency Medicine, Geneva University Hospitals, Geneva, Switzerland; Department of Pediatrics, Geneva University Hospitals, Geneva, Switzerland

## Abstract

**Background:**

To our knowledge no study has at the same time assessed patients’ satisfaction and their expectations concerning the organizational and contextual aspects of health care provided by their primary care physician (PCP). Assessing these aspects is important to inform future primary healthcare service planning. Our objective was thus to document patients’ satisfaction with and expectations from their PCP, in terms of availability and organization of their practices, and to assess whether these indicators varied across age groups and type of practice (solo, duo, group).

**Methods:**

Cross-sectional study based on the answers to questionnaires completed by patients consulting their PCP in Geneva, Switzerland. A random sample of PCPs was asked to recruit consecutively between 50 and 100 patients coming to the practice for a scheduled medical consultation. The patients were asked to complete an anonymous questionnaire centered on their satisfaction levels and expectations towards their PCP.

**Results:**

One thousand six hundred thirty-seven patients agreed to participate (participation rate: 97 %, women: 63 %, mean age: 54 years). Patient satisfaction was high for all the items, except for the availability of the doctor by phone and for the waiting time in the waiting room. The satisfaction rate increased with age and was higher for small practices. In relation to patients’ expectations from their doctor, older patients and patients visiting larger practices tended to be more demanding.

**Conclusions:**

Patients are generally highly satisfied with their PCP. They have a wide range of expectations which should be taken into account when considering potential improvements.

## Background

Primary care physicians (PCPs) are considered in many countries as a mainstay of the medical health system. Patient satisfaction is now regarded as an important indicator of health quality alongside quality of life, mortality and health costs [1, 2] and is of growing interest to health professionals and policymakers. It is a key factor in the global assessment of health care services, because patients and doctors do not always agree on the priorities to be given to different follow up indicators [3, 4] and patients’ satisfaction may influence their health status and medical costs [5]. The assessment of health care services using patients’ satisfaction is generally well accepted by PCPs [2]. Negative evaluations by their patients should lead doctors to reflect upon their practice in order to better respond to their patients’ expectations.

A European task force recently developed a standardized questionnaire, validated in French, in order to assess patient satisfaction in primary care, the Europep project (EUROpean task force on Patient Evaluation of general Practice) [6–8]. The questionnaire includes 23 questions assessing five dimensions of the patient-doctor relationship: relationship and communication, health care services, information and support, availability and access, organization. The satisfaction level is assessed for each question through a 5 points scale from poor to excellent. Several authors have used this questionnaire to assess patients’ satisfaction with medical health care services in primary care [7, 9–12]. These studies consistently showed a high degree of satisfaction among patients.

Primary care is experiencing new developments, mainly in terms of practice organization. Though a trend towards larger practices is currently noted in most high income countries, several studies have shown that patients seem to prefer small practices [9, 11, 13–18]. This finding could be explained by the fact that patients from small practices tend to report better accessibility of care, higher performance of receptionists and better continuity of care in the doctor-patient relationship [14]. However, defining the optimal size of practice is probably difficult, as small practices tend to show reduced quality performance [14].

To our knowledge, no study has at the same time assessed patients’ satisfaction and their expectations concerning the organizational and contextual aspects of health care provided by their PCP. Assessing these aspects is important to inform future primary healthcare service planning. Our objective was thus to document patients’ satisfaction with and expectations from their PCP, in terms of availability and organization of their practices, and to assess whether these indicators varied across age groups (<25 years, 25–65 years, > 65 years) and type of practice (solo, duo, group).

## Methods

### Recruitment of the doctors and the patients

This cross-sectional study took place in primary care practices in Geneva, Switzerland, in 2011. A random sample of 75 PCPs was selected from a sampling frame consisting of all the PCPs practising in the canton and members of the professional organisation of Geneva-based physicians. They were invited to participate by post, in order to include 25 PCPs in the study (expected participation rate: 33 %). In the absence of any response, the doctors were personally contacted by phone two or three weeks later.

### Data collection

A research assistant contacted each participating doctor’s medical assistant to inform them about the practical procedures for the study data collection. They were asked to recruit between 50 and 100 consecutive patients coming to the practice for a planned consultation. Patients were given oral and written information and, following written consent, were asked to complete a questionnaire containing general questions (age, sex, nationality, marital status, education, work status, health status) and questions about their satisfaction with and expectations from their PCP.

Patients’ satisfaction was assessed using six questions from the Europep questionnaire, validated in French, and scored on a 5 point Likert scale ranging from “poor” to “excellent”: helpfulness of staff, getting an appointment to suit the patient, getting through to the practice on the telephone, being able to speak to the doctor on the telephone, waiting time in the waiting room and providing quick services for urgent health problems [8]. Note that we included in the questionnaire only these six items regarding availability and access, because we were interested in assessing organizational aspects of health care provided by the PCPs.

Patients’ expectations were addressed using fifteen items: five items exploring accessibility and availability (waiting time deemed acceptable in the waiting room, waiting time deemed acceptable to get an appointment for urgent/non urgent health problems, to be able to speak to the PCP on the telephone, number of interruptions of the consultation related to phone calls deemed acceptable), two items regarding equipment (importance of having a laboratory/a X-ray equipment in the practice), three items exploring appearance and cleanliness (importance of wearing a white coat during the consultation, importance that the doctor washed his/her hands, importance of the cleanliness of the practice), and finally five items regarding accessibility and availability outside office opening hours (importance that the PCP is accessible by phone 24 h a day/the week-end during the day, importance that the PCP makes home visits/makes home visits 24 h a day/24 h the week-end during the day). These issues, identified through a review of the literature and discussion between the members of the research team, were selected as they were considered as the most important expectations to be studied. The questions concerning the importance given to equipment, appearance/cleanliness and accessibility/availability were assessed on a 5 point Likert scale ranging from “not at all important” to “extremely important”.

The questionnaire was pretested in a PCP’s practice (PS) and feedback was obtained from the respondents (*n* = 20), in order to estimate the mean time needed to complete the questionnaire and to identify any difficulties patients may meet in responding to the questions. The questionnaire was then modified accordingly. Finally, we pretested the new version of the questionnaire (*n* = 10). We also re-administered the same questionnaire to a small number of patients (*n* = 5) at 2 weeks interval to make sure the questionnaire was reliable in time.

Eligibility criteria were an age older than 15 years, understanding and writing French and having a planned appointment with the doctor; all the new patients and those suffering from disorders affecting their ability to consent were excluded. The self-administered anonymous questionnaire had to be completed in the waiting room of the practice, before or after the consultation, and deposited at the desk in a closed box. The research protocol was approved by the ethics committee for research in ambulatory care in Geneva (reference: 09–01).

### Sample size justification and statistical analysis

The sample size was estimated in order to measure the prevalence (50 %) of the « patient expectations » items (categorical data) with a margin of error inferior to 5 %. A sample size of 400 patients would have been sufficient, but we had to take the cluster effect into account, related to the fact that the patients were recruited in different practices (adjustment for taking into account the artificial decrease of the variance of measures collected in the same practice). Using an intra-class correlation of 0.025 (estimate based on published data and our personal experience), [19] and the assumption that 100 patients could be recruited in each practice, the inflation factor was 3.48 and our estimated total sample size was 1392 patients (400*3.48). In order to limit the PCPs’ workload for our study, we asked them to enroll between 50 and 100 patients during the study period. Thus, the number of PCPs needed was estimated to be between 14 and 28, leading to an average number of 21, which we rounded up to 25 to take into account possible withdrawals from the study.

Frequencies were used to describe binary and categorical variables, and means for continuous variables. For the items assessing satisfaction levels, we used the percentage of patients being satisfied or very satisfied (4 or 5/5 on the Likert satisfaction scale), as well as the mean score. We computed satisfaction levels in two different ways: we recorded the percentage of patients being satisfied or very satisfied (dichotomous variable), because this outcome was often reported in previous studies, as well as the mean score (continuous variable), because the additional information it contained might improve efficiency. The items assessing expectations were presented as categorical data or means, where appropriate. Subgroup analyses were undertaken for age groups (<25 years, 25–65 years and >65 years) and type of practice (solo, duo and group). For categorical data, we used Chi-squared tests to compare the frequencies obtained in each subgroup and conditional logistic regression to simultaneously adjust for other doctor and patient characteristics. For numerical data, we used one-way analysis of variance to compare the means in each subgroup and linear regression to control for confounding factors.

## Results

Among the 75 PCPs located in the Geneva area who were contacted at random, 31 % (*n* = 23) agreed to participate in the study (men: 61 %, mean age: 50 years), corresponding to 92 % of the expected number of participants. Twenty (87 %) were certified in general internal medicine, twelve (52 %) had their medical office located in a city (i.e. with a population greater than 15'000); the majority had solo or duo practices (39 % and 35 %), and almost two thirds employed medical assistants or administrative staff. On average, they were working 38.6 hours per week (SD 11.1), 4.7 days per week (SD 0.6), and were relatively experienced doctors (average number of working-years since certification: 10.5 (SD 10.1), average number of working-years in the current medical practice: 8.6 (SD 8.6). It is worth noting that the sample of 23 PCPs who agreed to participate seems to be representative of the study population (*n* = 650), as mean age (50 vs. 53 years) and sex (men: 61 % in the two groups) are similar.

One thousand six hundred thirty-seven patients provided consent to participate in the study, corresponding to 71 patients per doctor on average, well above the expected sample size (*n* = 1392). Only 45 patients refused to participate (women: 60 %, mean age: 64 years), the resulting participation rate being above 97 %.

Table 1 presents the patients’ socio-demographic characteristics. They were predominantly women (63 %), aged 54 on average (SD 18 years). They were divided into three subgroups according to their age: 6 % were under 25, 62 % between 25 and 65, and 32 % over 65. Half the patients were married, and three quarters were Swiss. Almost one third completed a university training or equivalent, and more than half had education beyond intermediate school. The majority had a professional activity (41 %) or was retired (30 %). Only 18 % rated their health as moderate or poor. Finally, the overall satisfaction level was judged excellent, since more than 95 % of the patients were very satisfied (i.e. having rated 4 or 5 /5 on the Likert scale).

**Table 1:** Patients’ socio-demographic characteristics (*n* = 1637)

Characteristics	n/N^a^	Percent
Female	981/1563	62.8
Age group		
< 25 years	97/1566	6.2
25 – 65 years	974/1566	62.2
> 65 years	495/1566	31.6
Marital status		
Single	386/1579	24.4
Married	783/1579	49.6
Divorced or separated	276/1579	17.5
Widowed	134/1579	8.5
Nationality		
Swiss	1163/1569	74.1
Italian	92/1569	5.9
French	89/1569	5.7
Portuguese	57/1569	3.6
Spanish	34/1569	2.2
Other (<2 %)	134/1569	8.5
Completed training		
No training	63/1505	4.2
Compulsory schooling	149/1505	9.9
Apprenticeship	506/1505	33.6
Baccalaureate or diploma from intermediate school	340/1505	22.6
University, FIT^b^, UAS^b^	447/1505	29.7
Work status		
Student	85/1569	5.4
Occupational activity	648/1569	41.3
Retired	467/1569	29.8
Recipient of unemployment or invalidity insurance	133/1569	8.4
Other (mainly house-wife/husband and without employment)	236/1569	15.1
General health status		
Excellent or very good	449/1571	28.6
Good	848/1571	54.0
Moderate or poor	274/1571	17.5
Number of consultations in the last 6 months		
1 - 2	676/1571	43.0
3 - 4	491/1571	31.3
5 - 6	266/1571	16.9
≥ 7	138/1571	8.8
Overall satisfaction level with the medical office		
1 (very low)	2/1584	0.1
2	8/1584	0.5
3	58/1584	3.7
4	438/1584	27.7
5 (excellent)	1078/1584	68.1

^a^ *FIT* Federal Institute of Technology, *UAS* = University of Applied Sciences

^b^The number of missing values per item varied from 53 to 132

Table 2 shows the satisfaction levels according to the three age groups, presented in two different ways: the % of patients very satisfied (i.e. having rated 4 or 5/5) and the mean score (SD). The vast majority of the patients were very satisfied, mainly concerning the overall satisfaction and the helpfulness of staff. However two items, the possibility to speak to the doctor by phone, and above all, the waiting time in the waiting room, were rated less favorably by the patients. Moreover, the satisfaction levels increased with age, regardless of the domain assessed (*p*-value for linear trend < 0.05 for all the items), though the association between age group and satisfaction items disappeared in multivariate analyses, except for being able to speak to the PCP on the telephone (*p*-value 0.02 for the dichotomous variable) and for providing quick services for urgent health services (*p*-value 0.02 for mean score).

**Table 2:** Patients’ satisfaction levels with the primary care physicians (PCPs) and their practices, according to patients’ age group

Characteristics	<25 y	25-65 y	>65 y	Crude *p*-value	Adjusted *p*-value^a^
Overall satisfaction level					
Very high to excellent satisfaction level, %	94.8	95.2	97.5	0.10	0.71
Mean satisfaction score (SD)	4.5 (0.6)	4.6 (0.6)	4.7 (0.5)	<0.001*	0.05
Helpfulness of the staff (other than the PCP)					
Very high to excellent satisfaction level, %	90.7	94.4	97.5	0.01	0.74
Mean satisfaction score (SD)	4.5 (0.7)	4.7 (0.6)	4.8 (0.5)	<0.001*	0.15
Getting an appointment to suit the patient					
Very high to excellent satisfaction level, %	87.6	91.2	95.0	0.01	0.92
Mean satisfaction score (SD)	4.3 (0.8)	4.5 (0.8)	4.7 (0.6)	<0.001*	0.42
Getting through to the practice on the telephone					
Very high to excellent satisfaction level, %	89.7	89.1	92.2	0.18	0.96
Mean satisfaction score (SD)	4.4 (0.7)	4.4 (0.8)	4.6 (0.7)	0.002*	0.43
Being able to speak to the PCP on the telephone					
Very high to excellent satisfaction level, %	66.7	79.7	88.2	<0.001	0.02
Mean satisfaction score (SD)	3.9 (1.0)	4.2 (0.9)	4.4 (0.9)	<0.001*	0.07
Waiting time in the waiting room					
Very high to excellent satisfaction level, %	56.7	75.5	80.6	<0.001	0.69
Mean satisfaction score (SD)	3.7 (1.0)	4.0 (0.9)	4.1 (0.9)	<0.001*	0.67
Providing quick services for urgent health problems					
Very high to excellent satisfaction level, %	78.3	90.0	93.7	<0.001	0.20
Mean satisfaction score (SD)	4.3 (0.9)	4.5 (0.7)	4.6 (0.7)	<0.001*	0.02

*p-value for linear trend < 0.001

^a^adjusted for all doctor and patient variables listed in Table 6

Table 3 presents patients’ satisfaction according to the type of practice. The satisfaction levels decreased with a higher number of PCPs in the practice, except for overall satisfaction, which showed similar ratings in the three types of practices, and satisfaction concerning the ability to speak to the doctor, which was highest for duo practices. In multivariate analyses, the differences in satisfaction between the three practice types remained statistically significant regarding helpfulness of the staff (for the dichotomous variable), getting through to the practice on the telephone, waiting time in the waiting room and providing quick services for urgent health problems.

**Table 3:** Patients’ satisfaction levels with the primary care physicians (PCPs) and their practices, according to type of practice

Characteristics	Solo	Duo	Group	Crude *p*-value	Adjusted *p*-value^a^
Overall satisfaction level					
Very high to excellent satisfaction level, %	95.3	96.4	95.2	0.54	0.48
Mean satisfaction score (SD)	4.6 (0.6)	4.7 (0.6)	4.6 (0.6)	0.04	0.87
Helpfulness of the staff (other than the PCP)					
Very high to excellent satisfaction level, %	96.5	96.0	92.6	0.01	0.02
Mean satisfaction score (SD)	4.7 (0.6)	4.7 (0.6)	4.6 (0.7)	<0.001*	0.15
Getting an appointment to suit the patient					
Very high to excellent satisfaction level, %	94.8	93.9	87.9	<0.001	0.42
Mean satisfaction score (SD)	4.6 (0.6)	4.6 (0.7)	4.4 (0.9)	<0.001*	0.35
Getting through to the practice on the telephone					
Very high to excellent satisfaction level, %	93.3	90.5	86.9	0.004	0.03
Mean satisfaction score (SD)	4.6 (0.7)	4.5 (0.7)	4.4 (0.8)	0.003**	0.02
Being able to speak to the PCP on the telephone					
Very high to excellent satisfaction level, %	83.6	87.0	73.3	<0.001	0.50
Mean satisfaction score (SD)	4.3 (0.9)	4.3 (0.8)	4.0 (1.1)	<0.001*	0.72
Waiting time in the waiting room					
Very high to excellent satisfaction level, %	81.9	76.4	71.2	0.001	<0.001
Mean satisfaction score (SD)	4.2 (1.0)	4.0 (0.9)	3.9 (1.0)	<0.001*	0.003
Providing quick services for urgent health problems					
Very high to excellent satisfaction level, %	94.9	91.5	85.1	<0.001	<0.001
Mean satisfaction score (SD)	4.6 (0.7)	4.6 (0.7)	4.4 (0.9)	<0.001*	0.02

*p-value for linear trend < 0.001, **p-value for linear trend < 0.05 and ≥ 0.001

^a^adjusted for all doctor and patient variables listed in Table 6

Table 4 shows patients’ expectations according to the three age groups. The younger patients were slightly more demanding in terms of waiting time in the waiting room, but less in terms of waiting period to obtain an appointment for urgent health problems. There was no age group difference for health problems viewed as non urgent by the patients, since the vast majority considered a waiting period of 5 days to two weeks as being acceptable. Many responders found it important to be able to speak to the doctor in the day, but the older patients were a little more demanding on this issue. Approximately two thirds, regardless of the age group, found acceptable that the doctor interrupt the consultation, once, because of phone calls. The laboratory and/or X-ray equipment were judged as being relatively important, mainly for the patients visiting the equipped medical offices. In addition, the older the patients visiting the equipped or non-equipped practices were, the more they considered the equipment important or not important respectively. Overall, wearing the white coat was judged less important. The same trend was seen again with increasing age, between the patients who consulted doctors wearing the white coat, and those who did not wear it. By contrast, two items, hand washing by the doctor and cleanliness of the practice, were a priority concern for all patients. Finally, neither having access to the doctor on the telephone 24 hours a day or on week-ends, nor receiving home visits, were judged as fundamental, but the older patients were a little more demanding on these issues. The association between age group and expectation items decreased in multivariate analyses, except for cleanliness of the practice, but remained statistically significant for several items (importance of wearing a white coat, importance that the PCP is accessible by phone 24 hours a day and makes home visits, importance of having X-ray for patients consulting unequipped PCPs).

**Table 4:** Patients’ expectations towards the primary care physicians (PCPs) and their practices, according to patients’ age group

Characteristics	<25 y	25-65 y	>65 y	Crude *p*-value	Adjusted *p*-value^a^
Waiting time deemed acceptable					
In the waiting room (%)				0.01	0.18
≤ 10 min	40. 6	26.7	27.6		
15 min	34.4	42.0	37.8		
20 min	7.3	10.3	13.6		
25 min	4.2	0.9	1.7		
30 min	10.4	16.6	16.3		
≥ 35 min	3.1	3.5	3.1		
To get an appointment for non urgent health problems (%)				0.09	0.69
In the day	2.1	6.9	9.3		
1 – 2 days	14.6	11.5	9.3		
3 – 4 days	20.8	17.3	14.4		
5 – 7 days	31.3	25.4	24.4		
1 – 2 weeks	20.8	25.7	27.3		
> 2 weeks	10.4	13.2	15.3		
To get an appointment for urgent health problems (%)				0.01	0.30
< 1 h	8.3	7.2	13.1		
In the day	48.5	53.7	48.4		
1 – 2 days	33.0	33.3	31.0		
≥ 3 days	10.3	5.8	7.5		
To be able to speak to the PCP on the telephone (%)				<0.001	0.06
No waiting time	7.3	6.9	13.8		
< 1 h	17.7	15.5	24.1		
In the day	57.3	58.2	49.5		
1 day	11.5	14.3	8.5		
≥ 2 days	6.3	5.2	4.2		
Number of interruptions of the consultation related to phone calls deemed acceptable (%)				<0.001	0.81
0	9.3	13.9	5.6		
1	66.0	63.7	65.8		
≥ 2	24.7	22.4	28.6		
Importance of having a laboratory in the practice (mean (SD))					
Patients consulting PCP with a laboratory	3.2 (1.2)	3.6 (1.5)	4.0 (1.3)	<0.001*	0.30
Patients consulting PCP without a laboratory	3.1 (1.2)	2.9 (1.6)	2.8 (1.7)	0.78	0.87
All the patients	3.2 (1.2)	3.4 (1.5)	3.8 (1.5)	<0.001*	0.40
Importance of having x-ray equipment in the practice (mean (SD))					
Patients consulting PCP with x-ray equipment	3.5 (1.1)	3.7 (1.4)	4.0 (1.4)	0.05**	0.43
Patients consulting PCP without x-ray equipment	2.8 (1.3)	2.2 (1.4)	2.1 (1.5)	0.01**	0.03
All the patients	3.1 (1.2)	2.8 (1.5)	2.8 (1.7)	0.07	0.10
Importance of wearing a white coat during the consultation (mean (SD))					
Patients consulting PCP wearing a white coat	2.6 (1.5)	2.8 (1.6)	3.1 (1.7)	0.001*	0.03
Patients consulting PCP not wearing a white coat	2.2 (1.4)	2.0 (1.4)	1.9 (1.4)	0.50	0.32
All the patients	2.4 (1.5)	2.5 (1.6)	2.8 (1.7)	0.01**	0.04
Importance of washing his hands (mean (SD))	4.7 (0.7)	4.6 (0.9)	4.5 (1.0)	0.17	0.29
Importance of the cleanliness of the practice (mean (SD))	4.5 (0.8)	4.6 (0.8)	4.7 (0.7)	0.04**	0.01
Importance that the PCP is accessible by phone 24 h a day (mean (SD))					
Patients consulting PCP accessible 24 h a day	2.1 (1.2)	2.1 (1.3)	2.5 (1.5)	0.03	0.10
Patients consulting PCP not accessible 24 h a day	2.2 (1.2)	2.1 (1.3)	2.3 (1.5)	0.05	0.09
All the patients	2.2 (1.2)	2.1 (1.3)	2.4 (1.5)	0.002**	0.02
Importance that the PCP is accessible by phone the week-end during the day (mean (SD)					
Patients consulting PCP accessible 24 h a day	2.4 (1.1)	2.1 (1.3)	2.3 (1.5)	0.05	0.34
Patients consulting PCP not accessible 24 h a day	2.2 (1.2)	2.0 (1.3)	2.1 (1.4)	0.52	0.19
All the patients	2.3 (1.1)	2.0 (1.3)	2.2 (1.4)	0.05	0.10
Importance that the PCP makes home visits (mean (SD))					
Patients consulting PCP accessible 24 h a day	2.2 (1.3)	2.9 (1.4)	3.5 (1.4)	<0.001*	0.01
Patients consulting PCP not accessible 24 h a day	2.4 (1.3)	2.3 (1.3)	3.1 (1.6)	<0.001*	0.65
All the patients	2.3 (1.3)	2.7 (1.4)	3.4 (1.5)	<0.001*	0.02
Importance that the PCP makes home visits 24 h a day (mean (SD))					
Patients consulting PCP accessible 24 h a day	1.6 (1.0)	2.6 (1.5)	2.6 (1.5)	0.10	0.56
Patients consulting PCP not accessible 24 h a day	1.7 (1.1)	1.6 (1.0)	1.8 (1.3)	0.001	0.33
All the patients	1.7 (1.1)	1.7 (1.1)	1.9 (1.3)	0.01	0.18
Importance that the PCP makes home visits 24 h the week-end during the day (mean (SD)					
Patients consulting PCP accessible 24 h a day	2.0 (1.0)	2.2 (1.2)	2.3 (1.5)	0.81	0.32
Patients consulting PCP not accessible 24 h a day	1.9 (1.1)	1.7 (1.1)	1.8 (1.3)	0.20	0.44
All the patients	1.9 (1.0)	1.8 (1.1)	1.9 (1.3)	0.09	0.40

*p-value for linear trend < 0.001, **p-value for linear trend < 0.05 et ≥ 0.001

^a^adjusted for all doctor and patient variables listed in Table 6

Table 5 presents patients’ expectations according to the type of practice. The patients visiting group practices were slightly more demanding in terms of waiting period to obtain an appointment, though the difference was statistically significant only for urgent health problems. They were also more likely to expect to be able to speak to the PCP on the telephone, and were more demanding in terms of equipment (laboratory and X-ray). The patients’ views regarding waiting time in the waiting room, hand washing and cleanliness of the practice, which were a priority concern for all the patients, doctor’s attire, doctor’s accessibility by phone outside practice hours and home visits, appeared to be relatively similar between the different practice types, although in univariate analyses some differences were statistically significant. In multivariate analyses the association with type of practice decreased for the vast majority of expectation items. However, there were statistically significant differences between practice types for waiting time in the waiting room and to get an appointment for urgent health problems (patients in solo practices expect to wait less time in the waiting room but longer to get an urgent appointment), white coat (patients in solo practices are more likely to prefer their doctor wearing a white coat in practices in which the doctor is wearing one, and more likely not to prefer this in practices in which the doctor does not wear a white coat), hand washing and home visits, as well as for importance of having a laboratory and X-ray facility for patients consulting unequipped PCPs.

**Table 5:** Patients’ expectations towards the primary care physicians (PCPs) and their practices, according to type of practice

Characteristics	Solo	Duo	Group	Crude *p*-value	Adjusted *p*-value^a^
Waiting time deemed acceptable					
In the waiting room (%)				0.16	<0.001
≤ 10 min	28.5	26.2	30.5		
15 min	42.1	40.4	37.0		
20 min	12.0	12.3	9.5		
25 min	1.1	1.0	2.1		
30 min	13.8	17.1	16.4		
≥ 35 min	2.5	3.0	4.6		
To get an appointment for non urgent health problems (%)				0.19	0.87
In the day	7.9	6.4	9.0		
1 – 2 days	9.2	12.2	12.2		
3 – 4 days	15.1	17.0	17.5		
5 – 7 days	24.7	23.2	27.1		
1 – 2 weeks	28.5	26.2	22.7		
> 2 weeks	14.6	15.0	11.6		
To get an appointment for urgent health problems (%)				0.01	0.01
< 1 h	7.7	6.9	13.3		
In the day	51.5	53.6	48.9		
1 – 2 days	34.5	32.4	31.0		
≥ 3 days	6.4	7.1	6.9		
To be able to speak to the PCP on the telephone (%)				0.01	0.39
No waiting time	9.9	7.9	10.6		
< 1 h	17.5	16.1	22.6		
In the day	57.5	57.8	48.7		
1 day	12.0	11.8	13.7		
≥ 2 days	3.2	6.4	4.4		
Number of interruptions of the consultation related to phone calls deemed acceptable (%)				0.01	0.71
0	9.3	11.3	11.7		
1	65.0	68.1	59.1		
≥ 2	25.7	20.6	29.2		
Importance of having a laboratory in the practice (mean (SD))					
Patients consulting PCP with a laboratory	3.3 (1.5)	3.9 (1.4)	3.8 (1.4)	<0.001*	0.21
Patients consulting PCP without a laboratory	3.5 (0.9)	2.5 (1.5)	3.9 (1.3)	<0.001*	0.01
All the patients	3.3 (1.5)	3.4 (1.6)	3.8 (1.4)	<0.001*	0.25
Importance of having x-ray equipment in the practice (mean (SD))					
Patients consulting PCP with x-ray equipment	3.3 (1.5)	3.3 (1.4)	4.0 (1.3)	<0.001*	0.18
Patients consulting PCP without x-ray equipment	2.2 (1.4)	2.2 (1.4)	2.4 (1.5)	0.50	0.004
All the patients	2.7 (1.6)	2.4 (1.4)	3.4 (1.6)	<0.001*	0.19
Importance of wearing a white coat during the consultation (mean (SD))					
Patients consulting PCP wearing a white coat	3.1 (1.7)	2.8 (1.6)	2.8 (1.6)	0.02**	<0.001
Patients consulting PCP not wearing a white coat	1.8 (1.2)	1.5 (1.0)	2.5 (1.7)	<0.001*	<0.001
All the patients	2.7 (1.6)	2.4 (1.6)	2.7 (1.7)	0.003	0.002
Importance of washing his hands (mean (SD))	4.7 (0.8)	4.6 (0.9)	4.5 (1.0)	<0.001*	0.01
Importance of the cleanliness of the practice (mean (SD))	4.6 (0.8)	4.6 (0.8)	4.6 (0.7)	0.87	0.10
Importance that the PCP is accessible by phone 24 h a day (mean (SD))					
Patients consulting PCP accessible 24 h a day	2.5 (1.5)	2.2 (1.4)	2.2 (1.4)	0.16	0.03
Patients consulting PCP not accessible 24 h a day	2.2 (1.4)	1.9 (1.2)	2.5 (1.5)	<0.001	<0.001
All the patients	2.3 (1.4)	2.0 (1.2)	2.3 (1.4)	<0.001	0.10
Importance that the PCP is accessible by phone the week-end during the day (mean (SD)					
Patients consulting PCP accessible	2.2 (1.4)	2.1 (1.3)	2.2 (1.4)	0.57	0.15
Patients consulting PCP not accessible	2.2 (1.3)	1.9 (1.2)	2.3 (1.4)	0.002	0.02
All the patients	2.2 (1.4)	1.9 (1.2)	2.2 (1.4)	<0.001	0.13
Importance that the PCP makes home visits (mean (SD))					
Patients consulting PCP accessible 24 h a day	3.3 (1.4)	2.9 (1.4)	3.0 (1.5)	0.001**	0.08
Patients consulting PCP not accessible 24 h a day	2.7 (1.4)	2.5 (1.4)	2.4 (1.5)	0.40	0.12
All the patients	3.2 (1.4)	2.9 (1.4)	2.8 (1.5)	<0.001*	0.04
Importance that the PCP makes home visits 24 h a day (mean (SD))					
Patients consulting PCP accessible 24 h a day	2.1 (1.4)	NA	2.5 (1.5)	0.40	0.65
Patients consulting PCP not accessible 24 h a day	1.8 (1.2)	1.6 (1.0)	1.6 (1.1)	0.03**	0.01
All the patients	1.8 (1.2)	1.6 (1.0)	1.8 (1.2)	0.003	0.14
Importance that the PCP makes home visits 24 h the week-end during the day (mean (SD)					
Patients consulting PCP accessible 24 h a day	2.2 (1.3)	NA	NA	NA	NA
Patients consulting PCP not accessible 24 h a day	1.8 (1.2)	1.7 (1.1)	1.9 (1.2)	0.02	0.22
All the patients	1.9 (1.3)	1.7 (1.1)	1.9 (1.2)	0.001	0.04

*p-value for linear trend < 0.001, **p-value for linear trend < 0.05 et ≥ 0.001

^a^adjusted for all doctor and patient variables listed in Table 6

Finally, Table 6 presents PCPs’ and patients’ characteristics which are simultaneously associated with the overall satisfaction level in multivariate analysis. The patients consulting uncertified doctors were less satisfied than those consulting certified doctors, when taking the other variables into account (OR 0.2, 95 % CI 0.1-0.6, *p*-value 0.003). The other characteristics were not associated with the overall satisfaction level in multivariate analyses.

**Table 6:** Adjusted associations between patients’ overall satisfaction towards the primary care physicians (PCPs) and their practices, and PCPs’ and patients’ characteristics using conditional logistic regression

Characteristics	OR	95 % CI	*p*-value
PCPs’ characteristics			
Women	0.8	0.2-2.6	0.71
Age	1.0	0.9-1.2	0.82
No certification	0.2	0.1-0.6	0.003
Urban medical office (>15,000 people)	0.8	0.3-1.9	0.56
Number of doctors practising in the medical office			
1	1.0 (ref.)		
2	1.2	0.4-3.3	0.77
≥ 3	1.0	0.3-3.4	0.98
Number of employees in the medical office			
1	1.0 (ref.)		
2	1.1	0.3-4.8	0.91
≥ 3	2.7	1.0-7.3	0.05
Number of days worked per week	1.2	0.3-4.7	0.78
Number of working-years since certification	1.0	0.9-1.1	0.88
Patients’ characteristics			
Men	1.0	0.5-1.9	0.95
Age group			
< 25 years	2.3	1.0-5.1	0.05
25 – 65 years	1.0 (ref.)		
> 65 years	1.3	0.2-9.4	0.81
Marital status			
Single	1.1	0.6-2.1	0.75
Married	1.0 (ref.)		
Divorced or separated	0.7	0.3-1.8	0.42
Widowed	1.2	0.3-5.9	0.81
Nationality			
Swiss	1.0 (ref.)		
Italian	0.8	0.3-2.8	0.76
French	1.8	0.3-9.7	0.51
Portuguese	0.6	0.2-2.0	0.42
Espagnol	1.2	0.2-6.8	0.87
Completed training			
No training	1.6	0.5-5.6	0.48
Compulsory schooling	0.5	0.1-1.5	0.21
Apprenticeship	1.0 (ref.)		
Baccalaureate or diploma from intermediate school	0.7	0.3-2.0	0.52
University, FIT^a^, UAS^a^	1.0	0.3-3.0	0.97
Work status			
Student	0.4	0.2-1.0	0.05
Occupational activity	1.0 (ref.)		
Retired	2.7	0.2-30.9	0.44
Recipient of unemployment insurance	1.2	0.5-3.0	0.65
Recipient of invalidity insurance	1.5	0.5-4.3	0.50
Excellent or very good general health status	1.6	0.8-3.3	0.17

^a^FIT = Federal Institute of Technology, UAS = University of Applied Sciences

## Discussion

Our study showed high patient satisfaction levels with organizational aspects of care, except regarding the possibility to speak to the doctor by phone and the waiting time in the waiting room, which were less well rated. The satisfaction ratings tended to increase with age but decrease with a higher number of PCPs in the practice. Only doctors’ certification status (specialist title holder or not) was associated with overall satisfaction in multivariate analyses.

Several studies used the questionnaire developed by the Europep project (EUROpean task force on Patient Evaluation of general Practice) to assess patients’ satisfaction with medical health care services in primary care. The results of these studies compare favorably with our own results. An international survey (ten countries including Switzerland, >1000 patients/country) showed a high degree of satisfaction among patients, especially in Switzerland, Germany and Belgium [7, 9]. A study carried out in Belgium (*N* = 994 patients, 42 PCPs) confirmed these results with more than 80 % of participants having rated 4 or 5/5 on the satisfaction scale for the great majority of the items [12]. In this study, questions about accessibility and availability were rated slightly less favorably, mainly helpfulness of the staff (79 % of the responders were satisfied or very satisfied) and the waiting time in the waiting room (60 %). Finally, according to a Slovenian study (*n* = 1809 patients, 36 PCPs) more than 80 % of the participants judged medical health care services very favorably [10]. Again, the lowest rating was given to the waiting time, since only approximately 60 % were satisfied or very satisfied. Although understandably many patients do not like to spend too much time in the doctors’ waiting room, this inconvenience seems less important to the patients than the free choice of doctor or appointment [20, 21]. In addition, time spent with the doctor seems a better predictor of the patients’ overall satisfaction than the waiting time spent in the waiting room [22]. In other words, the negative association between waiting time and patient satisfaction was found to be moderated by time spent with the doctor. As a result, the worst scenario was the combination of long waiting time and short visit time.

As seen in prior studies, [7, 10, 12, 17, 23, 24] we found that increased age was associated with improved satisfaction. It was hypothesized that older patients could receive more respect, consideration and attention from their PCPs and that they could be more reluctant to criticize them due to a strong doctor-patient relationship built up over the years [12]. Interestingly, whereas older patients reported higher satisfaction ratings, our study showed that they were also more demanding, suggesting, in spite of this, that their PCPs have met their expectations.

The place of primary care in the healthcare system is currently changing in many high income countries, including Switzerland, with a trend towards having larger practices with more staff. However, our results support previous studies, which have shown that patients seem to prefer small practices [9, 11, 13–18]. This finding could be explained by the fact that patients from small practices report better accessibility of care, higher performance of receptionists and better continuity of care in the doctor-patient relationship [14]. Another explanation may be that patients consulting PCPs working in large practices are more demanding, as suggested by our results assessing patients’ expectations. However, defining the optimal size of practice is probably difficult, as small practices tend to show reduced performance [14]. The impact on health costs, an important factor to be discussed, is not known and difficult to estimate. Larger practices may favor costs containment due to a reduction of unnecessary duplicated analyses and medical examinations, the patients’ medical files being available for all partners in the practice. However, in health systems based on a fee-for-service basis reimbursement (such as in Switzerland), doctors working in large practices, which are more often equipped with laboratory and X-ray facility, could be “encouraged” to expand service volumes. What is known is the influence of practice lists (i.e. the number of patients by doctor) on health costs, larger practice lists being associated with higher per patient costs [25]. This association could again have to do with the fact that an optimized practice organization enables larger treatment volumes.

Our study also extensively assessed patients’ expectations about the organizational aspects of the practice. In particular, the influence of age and practice size was explored. In summary, older patients and patients visiting larger practices tended to be more demanding, especially in terms of accessibility and availability, of professional attire (for older patients) and of equipment (for patients visiting large practices), though the association decreased for the vast majority of the items in multivariate analyses.

The finding of the influence of age on patients’ expectations could be explained by the fact that elderly persons often face complex and/or chronic medical problems, require a large number of medical consultations, including emergency and/or outside office opening hours health care. Interestingly, older patients were less demanding regarding waiting time in the waiting room, probably because they were predominantly retired and, as a result, had few time constraints. In addition, we found that patients consulting PCPs working in large practices were also more demanding, which could explain why they tended to report lower satisfaction ratings. We may interpret this finding by the fact that patient expectations of solo or duo practices could be more “modest” or, in contrast, that larger health care organizations attract a specific patient population which is more demanding.

We found that patients were generally more satisfied with PCPs equipped with a laboratory and/or an X-ray facility. These results are in agreement with other studies showing higher satisfaction levels with PCPs using point-of-care laboratory testing, which is increasingly used in primary care to manage patients with chronic or emergency conditions [26, 27]. This association probably reflects the fact that attending an outside laboratory would lead to extra time, transport costs and deferred feedback of the test results [27, 28]. Note that point-of-care laboratory testing is not only logistically simpler and economically more pragmatic, but seems to lead to the same or even better therapeutic control compared to usual laboratory testing [29, 30]. A study carried out in Switzerland confirmed its medical and economic utility, as it allowed a rapid management of the patient and avoided unnecessary additional consultations [31].

Few data are available to our knowledge about the satisfaction and/or expectations regarding management of the patients outside the office opening hours, and the studies were carried out in order to assess patients’ views related to the development and implementation of centers providing medical consultations [32, 33]. These studies tend to show that patient satisfaction is lower for telephone compared to face-to-face consultations.

Another dimension of patient satisfaction deals with appearance and cleanliness (white coat, hand washing and cleanliness). The association between white coat and satisfaction tends to be weak, but results from previous studies are conflicting [34–37]. As in our study, older patients and those usually consulting PCPs wearing the white coat tend to prefer doctors wearing the coat [35, 36, 38]. In-depth discussion of the findings regarding white coat is available elsewhere [39]. Finally, our study confirmed the results of several studies showing that hand washing by the doctor and cleanliness of the practice were a priority concern for all patients [40–43].

### Limitations

Since the study was carried out only in the Geneva area, our findings may not be generalizable to other regions of Switzerland and in particular to less urban and/or German or Italian speaking cantons. We excluded patients who consulted in an emergency situation or those who did not speak French. This may have introduced a selection bias since these patients are likely to have lower health or socio-economic status than the patients included in the study. This “selection” is reflected in the fact that the study participants presented a relatively favorable socio-economic status (they were predominantly Swiss, married, well-trained, working or retired, rating their health as good or excellent). However, the participation rate was high (>97 %) and patients were enrolled consecutively, thus reducing this risk of selection bias. Only 31 % of the doctors who were contacted agreed to participate, which may have introduced a selection bias since these doctors may have been more concerned with their patients’ level of satisfaction. The studied population is rather old as these patients are likely to consult more frequently due to their comorbidities which also increase with age. Thus, a consecutive sample will just reflect this age distribution. Finally, as the questionnaires were completed in the waiting room, patients may have been reluctant to be critical of their doctor, thus overestimating their satisfaction level. However the questionnaires were anonymous and the patients were clearly informed that their doctor would not have access to it.

## Conclusions

These findings highlight the relatively high satisfaction levels with PCPs in the Geneva region, confirming the results of studies carried out in other countries. They may inform healthcare providers about the influence of age and type of practice on patients’ satisfaction with and expectations from primary healthcare services. Finally, these findings are important to inform the changing primary healthcare picture as it moves away from the solo or duo model of practices, and embraces the new trend towards larger models of care.

## Abbreviations

PCP: Primary care physician
PCPs: Primary care physicians
Europep: EUROpean task force on Patient Evaluation of general Practice
SD: Standard deviation
FIT: Federal Institute of Technology
UAS: University of Applied Sciences
